# Match Day-1 Reactive Strength Index and In-Game Peak Speed in Collegiate Division I Basketball

**DOI:** 10.3390/ijerph18063259

**Published:** 2021-03-22

**Authors:** Adam J. Petway, Tomás T. Freitas, Julio Calleja-González, Pedro E. Alcaraz

**Affiliations:** 1 Washington Wizards Athlete Care Department, Washington, DC 20004, USA; 2 UCAM Research Center for High Performance Sport, Catholic University of Murcia, 30107 Murcia, Spain; tfreitas@ucam.edu (T.T.F.); palcaraz@ucam.edu (P.E.A.); 3 NAR-Nucleus of High Performance in Sport, São Paulo, SP 04753-060, Brazil; 4 Faculty of Sport Sciences, UCAM, Catholic University of Murcia, 30107 Murcia, Spain; 5 Department of Physical Education and Sport, Faculty of Education and Sport, University of Basque Country, UPV/EHU, 48940 Victoria-Gasteiz, Spain; julio.calleja.gonzalez@gmail.com; 6 Faculty of Kinesiology, The University of Croatia, 10110 Zagreb, Croatia

**Keywords:** neuromuscular, repeated jump, max speed

## Abstract

Basketball is a game of repeated jumps and sprints. The objective of this study was to examine whether repeated jump assessments the day prior to competition (MD-1) could discriminate between fast and slow in-game performances the following day. Seven NCAA Division I Basketball athletes (4 guards and 3 forwards; 20 ± 1.2 years, 1.95 ± 0.09 m, and 94 ± 15 kg) performed a repeated-hop test on a force platform before and after each practice MD-1 to assess Reactive Strength Index (RSI) and Jump Height (JH). Peak speed was recorded during games via spatial tracking cameras. A median split analysis classified performance into FAST and SLOW relative to individual in-game peak speed. Paired *T*-tests were performed to assess post- to pre-practices differences. An independent sample *T*-test was used to assess the differences between FAST and SLOW performances. Cohen’s d effect sizes (ES) were calculated to determine the magnitude of the differences. Statistical significance was set for *p* ≤ 0.05. Post-practice RSI and JH were significantly higher than pre-training values prior to the FAST but not the SLOW in-game performances. A significant difference was found for MD-1 RSI when comparing FAST and SLOW conditions (*p* = 0.01; ES = 0.62). No significant between-group differences were obtained in JH (*p* = 0.07; ES = 0.45). These findings could have implications on the facilitation of reactive strength qualities in conjunction with match-play. Practitioners should evaluate the placement of stimuli to potentiate athlete readiness for competition.

## 1. Introduction

Basketball is a court-based team sport that requires contributions from various physical parameters and bio-motor abilities [1]. These broad arrays of skills are principal components of in-game performance [2]. Particularly, basketball requires large expressions of speed and power qualities for match-play success. The technical and tactical aspects of the game put a high demand on the neuromuscular system relative to the sporting activity [3]. Therefore, the process of monitoring changes in these qualities for each individual player becomes paramount during the season (in view of the various stressors encountered by the players) [4,5,6], as it allows for evaluating longitudinal fluctuations over time [7] and provides insight on speed- and power-related performances. 

Within basketball, standardized and repeatable jumping assessments are amongst the most popular to assess neuromuscular function [8,9,10,11,12]. The ability to produce substantial amounts of force onto the ground to vertically displace the center of mass is an important skill contextually within the game, since basketball athletes execute around 45 jumps per game [3]. Thus, it is logical that practitioners collect and analyze jump data throughout the competitive season [13] to allow for a more in-depth neuromuscular function assessment [14,15,16]. This is particularly important since jump height (JH) alone does not always indicate athlete readiness as individuals may change movement strategies to achieve similar outputs [17]. In this context, the assessment of variables other than height in various jump tasks could be a suitable approach to monitor fatigue and readiness in the in-season period [18]. 

Previous research has examined jumping ability in basketball, with most studies utilizing the countermovement jump (CMJ) to assess neuromuscular function [18,19,20,21,22]. However, basketball mainly requires rapid stretch-shortening cycle (SSC) actions as well as the activation of H-reflex responses [23] that are not always reflected within the CMJ. To overcome this issue, repeated jumping and hopping tasks can be used, as they permit evaluating the ability to produce high vertical ground reaction forces in short ground contact times. In fact, the reactive strength index (RSI) (i.e., ratio of JH/contact time) has been previously used to measure both performance and fatigue within athletes [8,22]. 

Along with jumping, running speed is also an important characteristic in basketball [24]. Although the sport is played in a 28 by 15-m court, the ability to reach high top speeds and rates of acceleration can be extremely advantageous within the context of the game [1]. Whether it is via jumping- or running-based actions, athletes that can produce large amounts of force is a short amount of time are more likely to be in optimal positions on the court to garner competitive advantages (e.g., grab a rebound or intercept a pass) [25]. Conversely, if an athlete is producing less force and having longer ground contact times relative to their normative datapoint, this may be a potential sign of fatigue [26,27]. It is for this reason that examining the effects that fluctuations in reactive strength qualities have on the mechanical demands of in-game performance can provide informative decision-making on readiness to compete and recovery needs. 

To the best of the authors’ knowledge, no previous research has investigated whether a repeated-hop test performed the day before basketball competition can provide meaningful information regarding match-play mechanical demands. Therefore, the main purpose of this study was to investigate if fluctuations in reactive strength qualities could be used as an indicator to discriminate between faster and slower physical in-game performance the following day. This research may help coaches and sports scientists to make more informed decisions on both training and recovery. 

## 2. Materials and Methods

A prospective comparative study was conducted. Neuromuscular performance was assessed on the training day before competition (i.e., Match-day-1 [MD-1]) via a repeated-hop test. Match-play data was recorded during all 17 matches at the team’s home arena. All data was collected between the months of November 2017 and February 2018 by the strength and conditioning staff as routine for the daily assessment of fatigue and player loads. 

To evaluate neuromuscular performance (i.e., RSI and JH) on MD-1 for all 17 games, a repeated-hop test [28] was performed. The test was performed both pre-practice and post-practice to account for any of the acute effects imposed by the training session the day before the competition. A standardized warm-up of squats, lunges, and free arm swing CMJ preceded the assessment. Three repeated-hops were performed on a triaxial force platform (9260 AA-Kistler, Kistler Group, Winterhur, Switzerland) with the athletes’ hands on their hips. Players were instructed to jump as high and as fast as possible while spending minimal time on the plate without resetting between jumps. All tests were completed 15-min prior to, and after practice. The tests were disregarded if the athlete did not complete the standardized warm-up or did not fall within the 15-min windows. Likewise, data was not considered if the player did not test both pre- and post-practice. All jumps were recorded via a data acquisition system (DAQ System Type 5691 A- Kistler, Kistler Group, Winterhur, Switzerland). Each trial was exported to a TXT file and analyzed with the ForceDecks Software (Vald Performance, Brisbane, Australia) [29]. For each athlete, the difference between post- and pre-practice values were calculated (i.e., delta [Δ]). A positive or a negative integer would indicate an increase or decrease in neuromuscular performance, respectively. The mean of the 3 jumps RSI (calculated by dividing JH/contact time) in m∙s^−1^, and JH, in cm, were considered for analysis. 

Match-play activity profiles were tracked for each of the 17 home games throughout the 2017–2018 season via spatial tracking cameras (Sport VU^®^, Stats Perform, Chicago, IL, USA). This six-camera system was set up in the home gymnasium during competitions to track distance and speed of each athlete. The activity profile data was collected via Stats Sports VU software and exported to a customized spreadsheet (Microsoft Excel 2016, Microsoft Corporation, Redmond, WA, USA). The primary performance metric examined was peak speed (km∙h^−1^), given that it is an intensity-related variable that can provide a good gauge of neuromuscular readiness. A median split relative to individual’s peak speed was used to determine fast versus slow in-game performances. All 7 players competed in every home match. 

Data is presented as means and standard deviation. Data normality was tested using the Shapiro-Wilk test (*n* < 30). For every player, in-game performances (*n* = 17) were divided using a median split analysis into two groups (i.e., FAST: above the individual’s median value, and SLOW: below the player’s median) according to the peak speed achieved by each athlete during competition. Paired *T*-tests were performed to assess post- to pre-practices differences. An independent Sample *T*-test was used to assess the differences between FAST and SLOW performances. Cohen’s d effect sizes (ES) [30] were calculated to determine the magnitude of the differences and classified as: trivial (<0.2), small (>0.2–0.6), moderate (>0.6–1.2), large (>1.2–2.0), and very large (>2.0–4.0). Statistical significance was set for *p* ≤ 0.05.

## 3. Results

Table 1 shows the descriptive data and the comparison between FAST and SLOW performances. Post-practice RSI and JH were significantly higher than pre-training values prior to the FAST but not the SLOW in-game performances. Moreover, when considering the ergogenic response from before to after training (i.e., Δ), a significant difference was found for MD-1 RSI when comparing FAST and SLOW conditions (*p* = 0.01; ES = 0.62). No significant between-group differences were obtained in JH (*p* = 0.07; ES = 0.45). 

## 4. Discussion

The aim of the present study was to examine MD-1 pre- to post-practice differences (i.e., Δ) in repeated jump outputs and determine whether potentiation or degradation of neuromuscular performance in training could discriminate between faster and slower in-game physical performance. The main findings indicated that large gains in RSI (from before to after training) were observed the day prior to competitions in which higher peak speed values were reached during match-play. These preliminary results are novel and suggest that testing athletes’ repeated jump ability both prior to and after practice MD-1 (to account for any potential acute onset of fatigue or potentiation) can provide meaningful information regarding neuromuscular readiness to compete. This study is also unique in that it evaluated elite level basketball players throughout the entire competitive season. 

Of note, vertical jump has been previously found to be highly related to running speed [24] and a predictor of repeated-sprint ability in elite basketball players [25]. However, the present study is the first to identify what seemed to be a positive influence of gains in RSI MD-1 in peak speed of subsequent basketball competition. This finding could be extremely useful to practitioners considering that neuromuscular performance usually fluctuates during a typical in-season week [26]. Knowing that speed is a primary component in basketball [1,3], coaches can, therefore, optimize training strategies with the aim of maximizing reactive strength qualities prior to competition. This may, in turn, translate into superior neuromuscular status of the athletes that can place them at an optimal position for in-game success.

Remarkably, Δ JH MD-1 was not able to discriminate between FAST and SLOW in-game performances. Gathercole et al. [11] reported that neuromuscular function alternations 24 h after a fatiguing protocol were not detected when using JH alone (i.e., in both CMJ and drop jump tasks) and suggested that complementary variables such as Flight Time:Contact Time ratio should be assessed. Likewise, it appears that in the repeated-hop test herein, Δ RSI was more sensible than JH to determine neuromuscular readiness the following day. Based on the previous, it appears that an athlete’s ability to express high-force outputs in reduced contact times may better discriminate between FAST and SLOW games when compared to how high he can jump in a repeated-hop task. From a practical perspective, coaches are recommended to utilize the RSI metric obtained from a high rate of frequency test to assess their players on MD-1. 

The limitations of the present study should be addressed. Firstly, the small sample size limits the generalization of the current findings to other athletic populations. Nevertheless, since 17 games were analyzed here, the preliminary results obtained open a new perspective and should be investigated more in-depth. Secondly, it is important to keep in mind that peak speed is only one of many in-game physical parameters (e.g., accelerations, decelerations, or jumps); hence, further research should consider a more complete set of metrics to provide a clearer picture regarding match-play performance. Finally, variables other than RSI alone may influence subsequent in-game physical performance (e.g., MD-1 training load, recovery protocols, priming strategies). Thus, the reader should interpret the present results cautiously. 

In summary, MD-1 sessions that resulted in greater post-practice increases in RSI were observed prior to faster in-game performances when examining peak speed in elite collegiate basketball players. However, larger JH gains were not able to discriminate between faster and slower performances. These finding could impact stimuli provided to athletes prior to competition. Exposures to menu items that promote maximal high force outputs applied in reduced contact times may be most appropriate close to competition. 

## 5. Conclusions

Athletes with greater gains (i.e., Δ) in RSI from pre- to post-practice were found to achieve greater peak speeds in match-play the following day. Conversely, no differences were found between FAST or SLOW performances when JH was the variable analyzed. It is for this reason that professionals should closely examine acute adaptations to MD-1 as it may influence player selection or training strategies that place their athletes in the best position to succeed on the court. Having a critical thought process in regard to the sequencing of menu items is vital in the appreciation of the heterochronicity and different time courses of adaptive processes for varying stimuli. Specifically, actions that foster reactive strength and short ground contacts should be placed as close to the competition as possible within a training week. Further research on these topics is needed to gain a more robust insight into how to best create an environment for optimal neuromuscular outputs around match-play. The proper application of stimulus relative to match-play could have a direct impact on the optimization of neuromuscular status for in-game performance. 

## Figures and Tables

**Table 1:** Repeated-hop descriptive data from Match-Day -1 and comparison between FAST and SLOW in-game performances.

	In-Game Performance			
	FAST	SLOW	*p*	ES (95% CI)
Jump Height (cm)				
Pre-Practice	19.1 ± 5.7	20.9 ± 4.0	0.16	−0.37 (−0.9–0.16)
Post-Practice	23.5 ± 8.7 **	22.1 ± 4.5	0.45	0.20 (−0.32–0.73)
Δ	4.4 ± 8.1	1.2 ± 4.7	0.07	0.49 (−0.05–1.03)
RSI (m∙s^−1^)				
Pre-Practice	42.6 ± 20.1	45.1 ± 16.1	0.54	−0.13 (−0.66–0.39)
Post-Practice	57.5 ± 27.2 **	47.1 ± 17.4	0.16	0.45 (−0.09–0.98)
**Δ**	16.4 ± 27.1	2.0 ± 18.3	0.01	0.62 (0.06–1.17)

** Significant increase with respect to pre-practice (*p* ≤ 0.01). **Δ**: delta, change from pre- to post-practice; CI: confidence interval; ES: effect size; RSI: reactive strength index.

## Data Availability

The data presented in this study are available within the article.
